# Casein in the Blood; Synthetical Proof by Formation of Artificial Milk

**Published:** 1851-07

**Authors:** 


### ANATOMY AND PHYSIOLOGY.

*Casein in the Blood; Synthetical Proof by Formation of Artificial Milk.*—Dr. Panum, of Copenhagen, of whose interesting accounts of the discovery of Casein in the Blood, we gave a translation in the July number of this Journal for 1850, has published two additional articles on the subject in the *Bibliothek for Læger* for April and July 1850.

In his article in the April number, after recapitulating the facts brought forward in his previous paper, and pointing out the difficulty of establishing a chemical or a physiological distinction between casein and coagulated albumen, the author observes:

"If we institute a comparison between the substance precipitated from blood-serum by means of water and diluted acetic acid, and albumen separated in the same manner from its combination with soda, *we find* truly greater differences than can be distinguished between casein and coagulated albumen......

"Before the publication of my former contribution, I had already performed various experiments with the view of instituting a more accurate comparison between the material under consideration and albumen separated from its combination with soda. To procure albuminate of soda, I separated the albumen from the serum, then added soda, and applied slight warmth. A greater or less quantity of albumen (according to the quantity of soda employed) entered into combination with the alkali as albuminate of soda, and was precipitated by acetic acid. A difference was here observed between the albumen of serum and that of an egg. For while albumen from an egg required no warming to promote its entering into combination with soda, or its precipitation by means of acetic acid, the albumen of serum had to be slightly warmed before it would unite with an alkali, or give a precipitate with acetic acid.

"The albumen which was precipitated from albuminate of soda, as above described, was always *soluble with difficulty, even in a considerable excess of acetic acid;* eight or ten parts of acetic acid to one of albumen produced no solution. On the other hand, the material *ori,*

*ginally* present in the serum, and precipitated from it by acetic acid, was soluble in so slight an excess of acetic acid, that it is almost a *finer test of the acid, alkaline, or neutral properties of the liquid, than litmus paper*. This difference seems important; and can scarcely be without essential influence on the organic economy.

"The precipitate thrown down by acetic acid from albuminate of soda, appears as *firm flocks*, which are not changed by shaking, and which resemble what is seen when a not too highly diluted solution of albumen is boiled. The other matter produces a perfectly homogeneous opacity in the liquid, and only after some time forms something like a solid deposit in the bottom of the vessel, which, however, immediately disappears, and is resolved into a general opacity, when the liquid is slightly moved.

"Moreover, if the albumen precipitated from a somewhat diluted solution by boiling, or from a solution of albuminate of soda by acetic acid, be collected in a filter, it forms, when half dry, *an elastic mass, by no means glutinous*. The other substance, on the contrary, when half dry, forms *a very glutinous mass*, almost like turpentine or bird-lime.

"The albumen, when collected in a filter, has, when dry, a *dirty yellow brown colour*. In my previous contribution, I referred to the circumstance, that the substance I am describing sometimes assumed a *beautiful green colour* when thoroughly dried. At that time, I attributed it more to accidental circumstances than to any essential difference, that the substance became green or yellow-brown on drying, as from the same serum I sometimes procured a green and sometimes a yellow product I now find, that the substance is always of a beautiful green colour when the serum is perfectly clean and free from red blood corpuscles. But if serum be used, in which there is found a greater or less number of blood-corpuscles, the green colour is effaced by the accompanying redness of the blood, and the product becomes either dirty yellow or dirty brown, according to the number of blood-corpuscles. The green colour is removed neither by alcohol, ether, nor water, and seems to belong essentially to the casein.

"However lightly these differences may be thought of, they are yet not less important than the distinction already referred to, between the so-called soluble casein and albuminate of soda. The preconceived opinion that the material under consideration must be albuminate of soda can thus no longer serve as a proof against its identity with what is called casein, or the essential nitrogenous constituent of milk."

*Relative Quantity in the Blood.*—Dr. Panum states that he is engaged in a quantitative analysis of the casein in different individuals. He does not yet attempt to draw conclusions; but states that in 1000 parts of dried serum he has found in men, in 3 cases, from 4 to 7 parts; in 8 cases of women, from 5·5 to 12 5 parts. The greatest quantities (9·9—11·4—12·5) were in lying-in women shortly after parturition. In nurses there was an average of from 6·5 to 7·4 parts.

*Formation of Artificial Milk.*—In the July number of the *Bibliothek for Læger*, Dr. Panum writes as follows:—"It occurred to me that the question of the identity of the material I have describing with casein, might be solved by the *synthetical* method, which, though it can seldom be satisfactorily employed in scientific diagnosis, yet in the inability of analytical chemistry with regard to the protein compounds, is not to be despised. If it were possible, by adding the necessary constituents, to form from the substance under consideration some universally known product containing casein, such as milk or cheese, we should have a striking proof of the identity of the substance with casein.

I obtained a quantity of serum from bullock's blood. To this I added acetic acid, in the proportion of six drops of concentrated acid to an ounce of serum, and then a considerable quantity of water. The substance described in my former contributions sank, on standing, to the bottom of the vessel, so that the superabundant clear fluid could be poured off. On again adding water and letting the substance sink to the bottom, it was rendered almost perfectly free from soluble albumen, etc. After it had settled as much as possible, the water was poured off, so as not to waste the substance, which remained suspended in a greater quantity of water than casein is diluted with in milk. It was now my object to find out, whether the solution of this substance produced by salts or an alkali could be brought to coagulate by contact with the mucous membrane of the stomach of kittens or puppies; and whether it were possible to produce a substance which, in taste and other circumstances, should agree with milk. But as it would probably be difficult to make animals use the solution as milk, unless the other constituents of milk, namely butter and sugar, were mixed with it, and as the taste and peculiarities of cheese might be modified by them, I first tried, by adding these substances, to produce a liquid which should have some resemblance to milk. At a temperature of 30 Raumur, I added to the milky liquid phosphate of soda, till all the supposed casein was dissolved. I then added butter and sugar in the proportions in which they are usually contained in milk, and, after the butter was melted, I shook the whole mixture in a flask. The liquid, at first greyish or dirty yellow, became of the colour of egg-flip, and, as it cooled, became more and more like milk. The clearer and the less mixed with red blood-corpuscles the serum was, the clearer was the solution and the whiter the milk-like fluid. If the serum was perfectly free from blood-corpuscles, the emulsion, when diluted with water, had the same bluish colour as milk and water; but if there was a strong blood-red tinge in the serum, the solution in phosphate of soda had a reddish and the emulsion a weak yellowish tint. The product had a taste which bore a most striking resemblance to that of true milk; only it was a little more sweet, and left a feeble, though recognisable, bitter after-taste.

Under the microscope, I observed in the emulsion innumerable small globules, exactly like those in milk. On comparing them with those found in milk, scarcely any difference could be perceived, except that their average size in the artificial milk was somewhat greater than in the natural. The globules in the artificial milk were surrounded by a covering, and not simple drops of oil; this covering often showed a distinct though very fine folding, especially on the larger globules. The globules (like those of true milk) were not dissolved in ether, until the covering had been removed by treating them with nitric acid. But by the side of these small globules there were some which were somewhat greater, and presented in their interior appearances which, if they had been furnished from the organised body, would have been certainly considered as nuclei, granular cell-contents, etc.

As the difficulties, which might have been expected to arise in the microscopic comparison of the artificial and the true milk, had so unexpectedly disappeared, I felt myself called on to inquire whether any more perfect imitation could be attained. The most striking difference, which the artificial milk presented on a superficial examination, was that it required a far shorter time than real milk to form a layer of cream on the surface, and that, after standing some time longer, a layer of clear liquid was formed at the bottom of the vessel. I ascribed this failure to the greater average size of the globules in the emulsion, as compared with true milk-globules. When I employed sugar of milk in place of common sugar, the emulsion was uniform, and shewed no layer of clear liquid at the bottom of the vessel, even when more water was added. At the same time, the average size of the corpuscles became less, so that they could not be distinguished by their size from the true milk-globules, and they were now free from any appearance of nuclei or granular cell-contents. The cream was longer in rising to the surface; and the great sweetness which the emulsion had, when made with common sugar, was no longer prominent, so that the liquid tasted still more like milk. A slight smack of salt disappeared, when the butter was washed in water; and a slightly harsh taste of the butter was still farther removed when unsalted butter was used, which had been freshly churned, and washed with water. There was still a slightly bitter after-taste, but this was removed by using carbonate instead of phosphate of soda; a much smaller quantity of the former salt being required to produce the required solution. It was now impossible for me to recognise, by the taste, any difference between artificial and real milk. As, however, it might be said that "*de gustibus non est disputandum*," I submitted the liquid to the taste of others, with the same result.

It now only remained, to render the agreement perfect between the artificial and true milk, that the former should coagulate when brought into contact with the mucous membrane of the stomach. When I used common sugar, it was not wonderful that my attempts thus to make the liquid coagulate, failed; for it is well known, that the addition of sugar to milk retards or prevents its coagulation. But, even after I had used sugar of milk, I could not perfectly succeed in making the milk coagulate. Instead of fresh calf's stomach, I used the artificially prepared swine's stomach, which is commonly used in the dairies, with or without a temperature of 40 R.; and I also gave the liquid to two kittens, who drank it greedily, and whom I then killed. Under these circumstances, as well as by mere standing, the artificial milk turned, and separated into a clear liquid, and a thick curdy mass, which had very strongly the well known smell of sour milk; but I could not produce a consistent coagulum, which could be collected in linen, and pressed out. But when the original substance separated from the serum, after being washed from soluble albumen, was warmed, it formed masses resembling pressed curds, and could be collected on a cloth, while the liquid ran through quite clear.

The identity of artificial with natural milk, and of the substance found in the blood with casein, may seem to be not proved; yet the resemblance is so great as to strengthen the probability of their being identical. I have in vain endeavoured to produce from albumen, precipitated from albuminate of soda by acetic acid, and dissolved in phosphate or carbonate of soda, a liquid having the slightest resemblance in taste to milk.—*London Journal of Medicine.*

